# Feasibility of the Autism Navigator^®^ JumpStart to Coaching in Everyday Activities course in South Africa

**DOI:** 10.1177/13623613231223784

**Published:** 2024-02-08

**Authors:** Nola Chambers, Petrus J de Vries, Amy M Wetherby

**Affiliations:** 1 University of Cape Town, South Africa; 2 Florida State University, USA

**Keywords:** Autism Navigator, autism spectrum disorder, early intervention, Early Social Interaction, implementation, non-profit sector, professional development, South Africa

## Abstract

In low-resource settings, non-profit organisations play an essential role in providing services and support for families with young children with autism. However, resource constraints limit access to quality training in evidence-based interventions. Web-based training may help fill this gap. This study examined the feasibility, acceptability and appropriateness of the web-based Autism Navigator^®^ JumpStart to Coaching in Everyday Activities course for 26 non-profit organisation providers (10 specialists and 16 non-specialists) in South Africa. Quantitative and qualitative methods were used including a post-training survey. Sixteen providers (62%) completed the course over an average period of 5.58 months, with many completing the course during lockdown restrictions, suggesting time as a barrier to course completion. No provider characteristics were related to attrition, nor to the time taken to complete the course. Better English proficiency, higher education levels and more positive attitudes to implementing an evidence-based intervention were associated with the number of attempts needed to pass some learner assessments, and to perceptions of course feasibility, acceptability, and appropriateness. Adaptations in the training delivery (using a group format and local group conference calls) and inclusion of South African video clips were identified as potential facilitators to acceptability and appropriateness.

**Lay Abstract:**

In low-resource settings, non-profit organisations play an essential role in providing services and support for families with young children with autism, including in Africa. However, non-profit organisation service providers may not have access to quality training in proven intervention methods. Web-based or online courses may help to meet this need. In this study, we invited a group of specialist (10) and non-specialist (16) non-profit organisation providers in South Africa to complete a web-based course, Autism Navigator^®^ JumpStart to Coaching in Everyday Activities, a 20-h self-paced course that provides training in an evidence-based parent coaching intervention called Early Social Interaction. We evaluated acceptability, appropriateness, and feasibility of the training. Of the 26 who enrolled, 16 completed the course (7 specialists and 9 non-specialists). All providers found it difficult to find time to do the course until the lockdown restrictions due to COVID-19, when most completed the course. Those whose first language was not English experienced more difficulties with two of six learner assessments and those who were not clinical specialists had more difficulty with the coaching strategies learner assessment. Most providers rated the course highly feasible, acceptable, and appropriate stating that the course content was very valuable and helpful in equipping them to serve their families. They felt the extensive video clips and regular meetings with a local trainer helped them engage with and understand the material. They suggested that including South African video clips would make the course more relatable. The fact that the course was web-based was identified as a strong benefit, especially during COVID-19 restrictions.

## Introduction

### Contextual setting and the non-profit sector

Recent research suggests that there is a significant treatment gap for children with autism in South Africa (Pillay et al., 2022). South Africa is considered a developing or emerging upper-middle income economy, but remains a dual economy with one of the highest inequality rates in the world (World Bank, 2018). Most of the population live in informal housing, participate in an informal economy and rely on the public or non-profit sector for education, healthcare and transport. Although there is a comprehensive public healthcare system, most services for autism consist of one session per month or less (Pillay et al., 2022). Consistent with calls for implementation efforts across the globe (Boyd et al., 2022; Stahmer et al., 2019; Vivanti et al., 2018), this treatment gap requires systematic efforts at feasible and sustainable training in evidence-based interventions and implementation of these interventions within existing systems of care (Franz et al., 2017, 2018; Lord et al., 2022).

One such existing system of care in South Africa is the non-profit sector. Non-profit organisations (NPOs) often fill important gaps in public service delivery, particularly in low-resource settings. A recent review in South Africa found that NPOs provide critical services in health and wellness, socialisation and education spheres of society and assist citizens who may not easily access government services (Choto et al., 2020). There are numerous local, regional and national NPOs in South Africa that serve families with children with autism, either exclusively or as part of a broader service to families of children with disabilities. Staff generally include a mix of clinical specialist and non-specialist staff, allowing for mentoring and task shifting. Direct family services, including in-person home visits, are often one part of their service delivery mandate, in addition to raising awareness, advocacy, training and developing community resources. The inclusion of in-person home visits in their service delivery mandate suggests NPOs as a good fit for providing home-based parent coaching interventions for children with autism. However, access to training in these evidence-based interventions for providers in NPOs is limited.

### Evidence-based interventions for young children with autism

There is a growing evidence base for the positive effects of naturalistic, developmental, behavioural interventions (NDBIs) for young children with autism (Crank et al., 2021; Sandbank et al., 2020). NDBIs refer to a family of interventions with common elements including that they occur in the natural environment, with natural communication partners, and intervention goals are informed by developmental theory (Schreibman et al., 2015). One example of an evidence-based NDBI is the Early Social Interaction (ESI) model, designed as a caregiver-mediated intervention for toddlers with autism (Wetherby & Woods, 2006). The intervention aims to improve children’s active engagement in their natural everyday routines at home by coaching caregivers to implement a menu of transactional supports. This intervention has shown strong evidence of effectiveness when implemented by trained specialists in the United States (Guthrie et al., 2023; Wetherby et al., 2014, 2018). ESI incorporates a collaborative coaching model where provider and parent build consensus to identify everyday activities, goals and supports to achieve these goals (Lorio et al., 2020; Wetherby & Woods, 2006).

In order to promote wider scale dissemination and implementation of ESI, and similar to other NDBI developers (Wainer & Ingersoll, 2013), the developers incorporated mobile technology to create a comprehensive professional development solution known as Autism Navigator^®^ (Fingert et al., 2018; Wetherby et al., 2018). This consists of a suite of web-based training tools and courses, with tiers to certification designed with an implementation focus. In the Autism Navigator JumpStart to Coaching in Everyday Activities (JumpStart) course, the essential elements of the ESI model have been packaged into a 20-h web-based, self-paced course that systematically incorporates adult-learning principles (Myers et al., 2022), such as learner assessments, practice exercises, and interactive slides with illustrative videos and audio narration to engage learners in the material. Further information about the ESI model and course content can be found in the Supplemental Materials. Importantly, the JumpStart course is designed to be more than a ‘one-time-only’ course, but rather as an ongoing resource with an intervention fidelity manual for providers to refer back to and explore as they are learning how to implement ESI with each new family and as they need ideas to address new challenges that arise in their practice.

The ESI intervention model and the web-based training model of the Autism Navigator suite of courses and tools is a potentially good fit to meet the significant training needs of NPO providers in South Africa. The web-based learning model of the JumpStart course could have a potential reach that in-person training approaches cannot achieve. There is a so-called ‘digital divide’ in low- and middle-income countries (LMICs) (World Bank, 2016) that could impact feasibility of web-based professional development courses. For example, in the nursing field, limited computer access and limited or slow Internet connections have been listed among a range of barriers to online continuing professional development (Feldacker et al., 2017; Hosey et al., 2016; Makombe et al., 2019). However, Kumm and colleagues recently found that of all the technological innovations in the autism field, those that can be accessed by computer or mobile phone, such as the JumpStart course, are considered to have high overall feasibility in LMICs in terms of accessibility, acceptability, scalability and sustainability (Kumm et al., 2022). Consistent with this, we found that another Autism Navigator course, Autism Navigator for Primary Care, which focuses on the early signs of autism, was found to be feasible, acceptable and appropriate by a wide range of South African service providers in the educational, private and public health, and non-profit sectors (Chambers et al., 2018).

### Implementation barriers in low resource settings

Beyond access to technological infrastructure, there are a range of contextual aspects that may impact the feasibility of a training such as the JumpStart course with providers in a new context, like NPO providers in South Africa (Aarons et al., 2017; Feldacker et al., 2017). Specifically, individual provider characteristics may impact the feasibility of online continuing professional development (CPD) training. In South Africa, there are 12 official languages, and hence, English proficiency may play an important role in the ability to benefit from English-language CPD courses (Chambers et al., 2018). Educational level has been associated with completion of online courses generally (Reinwand et al., 2015). Finally, attitudes to implementing a new evidence-based practice described in a training may impact the benefit received from the training itself (Aarons, 2004; Aarons et al., 2012). Such attitudes have been included in the Autism Model of Implementation as an important provider characteristic to assess in implementation efforts (Drahota et al., 2012, 2021).

These provider characteristics have all been identified as important ‘inner context’ factors in the Exploration, Adoption/Preparation, Implementation and Sustainment (EPIS) framework (Aarons et al., 2011), which has been used for guiding exploration of the many factors that may impact the perception and implementation of an evidence-based training or intervention for children with autism at a community level (Stahmer et al., 2019). In terms of this framework, we considered the web-based JumpStart course as a *bridging strategy* to overcome contextual limitations in access to training. We also implemented extensive stakeholder engagement prior to enrolment to promote implementation success. We were particularly interested in *inner context* factors related to the provider characteristics mentioned above that might impact the experience and perceptions of the feasibility, acceptability and appropriateness of the JumpStart course and ESI intervention model in South Africa.

The aim of this study was to evaluate the feasibility, acceptability and appropriateness of the web-based Autism Navigator JumpStart to Coaching in Everyday Activities course to train specialist and non-specialist NPO providers in an evidence-based, caregiver-mediated early intervention for children with autism and to determine whether measures of feasibility, acceptability and appropriateness were related to a range of demographic variables including education level, self-rated English proficiency and attitudes to implementing an evidence-based intervention.

## Methods

### Ethical considerations and study design

This study was approved by the Human Research Ethics Committee (HREC) at the University of Cape Town, protocol number: 682.2017. A single group descriptive design using mixed methods was used.

### Participants

NPO providers were recruited from NPOs (1) with a mandate to serve families affected by autism as their sole focus, or part of a broader service to families with young children, (2) offered home visits as part of their service delivery model and (3) were willing to complete the training. There were no restrictions on provider participant age, education level, first language or clinical expertise for recruitment into the study. NPO managers were asked for permission to invite providers willing to complete the training but it was not made mandatory in any of the NPOs. We expected that within each NPO one or more providers may become the ‘caregiver coaching champion’ later responsible for implementing or promoting the intervention, but we allowed all interested providers to participate as a capacity-building initiative and to build relationships with the NPOs, even those who might not ultimately implement the intervention themselves. We acknowledge that this could have had an impact on completion rates in the study.

### Provider enrolment and demographics

Between September 2019 and September 2021, 26 NPO providers enrolled on the JumpStart course. These providers were recruited from five NPOs in South Africa. Two of these NPOs had an exclusive autism-specific service delivery mandate, while the other three served families with a variety of needs, including autism. The sample included 16 non-specialists (defined as those without a clinical degree) and 10 specialists (defined as those with a clinical degree, including 5 social workers, 3 speech-language therapists and 2 experienced autism intervention providers and training coordinators with bachelor’s and master’s degrees).

The age of the sample ranged from 25 to 66 years (mean: 39.77; SD: 10.90) and all providers were female. The sample was roughly equally divided into black African (n = 14) and white (n = 12) participants. Just over half spoke English as a first language (n = 16) with a range of other African first languages, including isiXhosa (5), isiZulu (1), Setswana (2), SeSotho (1) and Afrikaans (1). Many reported only learning English after 5 years of age (n = 11). Most participants rated their comprehension of English as good or excellent (n = 24), with two rating their English comprehension as average. A small proportion (n = 5) reported not owning their own computer and four claimed never to have completed any online course prior to the study. Most had received previous training on intervention with children with autism (n = 22), but there was variation in the amount of previous experience working with families (17 had less than 5 years’ experience). A large proportion of the sample reported feeling ‘somewhat confident’ (n = 13) or ‘not confident’ (n = 5) working with very young children with autism.

### Procedures

#### Stakeholder engagement

Prior to participant enrolment, time was spent meeting with local and national NPOs that serve families with young children with autism to discuss their service priorities and service delivery models and describing the training course and intervention model to determine whether it would be a good fit for the organisation. Prior to COVID-19 restrictions, this took the form of in-person stakeholder meetings which shifted to virtual meetings from April 2020.

#### Participant enrolment

Once organisational approval and consent was obtained from interested NPO directors, NPO staff were invited to participate by the chairperson or director of the organisation. All staff within the NPO were encouraged to participate, but it was not required by the NPO director.

#### Course enrolment and completion

Once provider participants signed their consent to participate, they completed two pre-training measures (demographic form and attitudes to implementing an evidence-based intervention; see section ‘Measures’). Each participant was then sent an enrolment code for the course. Participants created their own account on the Autism Navigator learning platform and began the course. Participants were encouraged to complete the course within 3 months to build momentum and aid retention, although rate of completion in the United States is typically around 6 months (Wetherby, personal communication, 4 November 2022). Bimonthly group conference calls were scheduled with the individuals or groups from each organisation to discuss the information they had learned in the preceding 2 weeks and answer questions. Participants were also encouraged (but not required) to attend the monthly Autism Navigator Knowledge and Skills webinars hosted by the Autism Navigator team in the United States. This was not made a requirement as the webinars took place outside of work hours for the providers in South Africa. The South African group calls were a local adaptation of the training in South Africa and were designed to take the place of these international webinars. Participants were not remunerated for their time spent doing the training or attending conference calls. Following completion of the course, participants completed a post-training survey. The pre- and post-training forms were completed using REDCap (Research Electronic Data Capture), a secure, web-based software platform designed to support data capture for research studies (Harris et al., 2009, 2019). Electronic data collection was essential for this study as provider participants were located across all nine provinces of South Africa.

### Measures

#### Demographic questionnaire

The provider questionnaire obtained information on the providers’ age, gender, ethnicity, first language, educational level and current scope of practice. As the JumpStart course requires access to suitable technological infrastructure, additional information on access to and self-reported proficiency with technological devices and online learning were also probed.

#### Attitudes to using a new evidence-based intervention

Providers’ attitudes to implementing a new evidence-based practice were measured before starting the course using the Evidence-based Practice Attitude Scale-36 (EBPAS-36) (Rye et al., 2017). The EBPAS has been used widely in the field of implementation science, with limited research in South Africa using the original 15-item version (Booysen et al., 2019; Padmanabhanunni, 2018). It is thus not specific to the JumpStart course. The 36-item version was used in this study to provide a more comprehensive understanding of attitudes to evidence-based practices among this diverse sample of NPO providers (Rye et al., 2017). The EBPAS-36 has shown adequate psychometric properties with regard to reliability, construct validity, cross-cultural validity and has also shown promise as a pragmatic tool for use in real-world contexts (Rye et al., 2017). It yields a total score indicating overall attitude, as well as 12 subscale scores relating to Requirements, Appeal, Openness, Divergence, Limitations, Fit, Monitoring, Balance, Burden, Job Security, Organisational support and Feedback. Responses to statements are given on a 5-point Likert-type scale from 0 (not at all) to 4 (to a very great extent). To reduce bias, items from five subscales are negatively framed and are reverse scored before calculating the total score (Divergence, Limitations, Monitoring, Balance and Burden). Higher scores indicate more positive attitudes. We used the total score in our analyses in this study. Cronbach’s alpha for the total score was 0.77 for this sample.

#### Course utilisation (feasibility)

Measures of course utilisation included rate of course completion (where course completion was defined as having opened all slides within the four Guide Books, two Field Guides and two Video Libraries), length of time taken to complete the course and mean number of attempts required to pass the six learner assessments, defined as a score of >80%.

#### Post-training survey (feasibility, acceptability and appropriateness)

Following course completion, participants completed a short pragmatic quantitative measure of course acceptability, appropriateness and feasibility adapted from Weiner et al.’s (2017) Acceptability of Intervention Measure (AIM), Intervention Appropriateness Measure (IAM) and Feasibility of Intervention Measure (FIM). These open-access, pragmatic scales were developed specifically to address a quantitative measurement gap in the implementation science literature, with clear definitions for each construct. The scales were also designed to allow researchers to individualise the items to specify the treatment or innovation under study, in this case, the JumpStart training course. Definitions of each construct and an example item used in this study are as follows:

- Acceptability: perception of the innovation or intervention as agreeable, palatable or satisfactory (e.g. the Autism Navigator training is appealing to me);
- Appropriateness: the perceived fit, relevance or compatibility of the innovation or intervention for their service setting, and/or perceived fit of the innovation or intervention to address a specific problem (e.g. the Autism Navigator training seems fitting);
- Feasibility: the extent to which the innovation or intervention could be successfully used or carried out within their service setting (e.g. the Autism Navigator training seems doable).

Each construct is measured using 4 items, for a total of 12 items which were presented in randomised order in this study. Items required responses on a 5-point Likert-type scale ranging from completely disagree (1) to completely agree (5) and higher scores indicate more positive perceptions of the JumpStart course. The scale has a Flesch reading ease score of 95.15 which is a grade 5 reading level (Weiner et al., 2017). An open-ended item was added at the end to solicit open-ended feedback about the course. Cronbach’s alpha for each scale was very high in this sample: 0.97 (acceptability), 0.96 (appropriateness) and 0.95 (feasibility).

### Data analysis

Descriptive statistics were used to summarise participant demographic characteristics, the EBPAS-36, course utilisation and completion measures, and the post-course survey. Non-parametric (Spearman rank) correlations were calculated to examine relationships between demographic characteristics (English proficiency, education level and attitudes) and course utilisation/completion and perceptions of feasibility, acceptability and appropriateness. Open-ended feedback was analysed using content analysis to identify themes relating to feasibility, acceptability and appropriateness.

#### Community involvement

Extensive stakeholder engagement was implemented in the search for study participants. NPO directors and providers engaged in numerous meetings with the research team to discuss if and how the JumpStart training course and intervention model would fit their needs, preferences and service delivery models. The bimonthly conference calls were included directly in response to the preferences of the NPOs. The benefits of receiving the training and support were intended to last beyond the duration of the study and enhance quality of service delivery in these settings.

## Results

### Feasibility of the JumpStart course

#### Course completion

In the 2-year project window, 16 of the 26 enrolled completed the course (62%). Of the remaining 10 ‘incomplete’ participants at the end of the study period, 3 had enrolled shortly before the end of the study period and were still in the process of completing the course (all specialists), 2 had left their organisation before completing the course and had abandoned the course thereafter (non-specialists) and 5 were still at the participating organisations but had not responded to numerous follow-up communications and were considered ‘course abandoned’. These five participants were all non-specialists. Relationships between provider characteristics and whether the course was completed or not and the time taken to complete the course are provided in Table 1. No provider characteristics distinguished those who completed the course from those who did not.

**Table 1.** Impact of provider characteristics on course completion (n = 26) and time taken to complete the course (n = 16) as indicated by Spearman’s rho correlation coefficients rho; p-value.

	Whether the course was completed (n = 26)	Rate of course completion among the completers (n = 16)
Self-rated English proficiency	0.26; (p = 0.20)	0.01; (p = 0.96)
Education level	−0.07; (p = 0.75)	0.25; (p = 0.35)
Access to own computer	0.02; (p = 0.94)	0.23; (p = 0.40)
Previous experience completing on online training	0.06; (p = 0.77)	−0.02; (p = 0.94)
Attitudes to implementing a new evidence-based practice	−0.20; (p = 0.33)	−0.09; (p = 0.77)

#### Time taken for completion

On average, the 16 completers took 5.58 months (SD: 3.78) to complete the course, with a wide range extending from 1.53 to 13.97 months. Similar to analyses regarding course completion, no provider characteristics were significantly related to the time taken to complete the course among the 16 completers (see Table 1).

#### Learner assessments

Table 2 summarises the mean number of attempts needed by the group to obtain a score of 80% on each of the learner assessments, as well as the proportion of participants requiring more than one attempt to pass the six learner assessments contained in the course. The size of sample differs for each learner assessment as not all providers completed the learner assessments. About a quarter of the group needed more than one attempt to score 80% on most learner assessments, other than for Guide Book 1, where just under half required more than one attempt. One provider took the Guide Book 4 learner assessment 18 times in order to achieve the required 80%. This provider’s comments during conference calls suggested this was due to difficulties with the language complexity of the multiple-choice questions. This was confirmed by significant Spearman rank correlation coefficients between self-rated English comprehension and the number of attempts needed to pass the learner assessments on Guide Books 3 and 4 (see Table 2). There was also a significant relationship between educational level and number of attempts to pass the learner assessment of Field Guide 2, where those with higher educational levels (and hence specialist clinical degrees) required fewer attempts to pass the learner assessment focused on the clinical skills of parent coaching. Attitudes to implementing a new evidence-based practice were inversely related to the number of attempts to pass learner assessment for Guide Book 3 and showed a large but non-significant inverse relationship with attempts for Field Guide 1. These suggest that more positive attitudes were related to fewer attempts needed to score 80%.

**Table 2.** Attempts needed to pass the learner assessments and relationship with provider characteristics.

	Mean (SD) attempts needed to score 80%	n requiring >1 attempt to pass learner assessment	Range in number of attempts to score 80%	Spearman correlation coefficients rho (p-value)		
				Self-rated English comprehension	Education level	Attitudes (EBPAS total score)
Guide Book 1 (n = 24)	2.37 (2.10)	11 (46%)	1–9	0.36 (p = 0.08)	−0.46 (p = 0.02)	−0.22 (p = 0.30)
Guide Book 2 (n = 25)	1.72 (1.79)	5 (20%)	1–9	0.31 (p = 0.13)	−0.34 (p = 0.10)	−0.30 (p = 0.15)
Guide Book 3 (n = 23)	1.74 (1.69)	5 (22%)	1–7	**0.42 (p = 0.04)**	−0.38 (p = 0.07)	−**0.47 (p = 0.02)**
Guide Book 4 (n = 20)	2.55 (4.20)	7 (35%)	1–18	**0.61 (p = 0.04)**	−0.03 (p = 0.91)	−0.25 (p = 0.28)
Field Guide 1 (n = 15)	1.33 (0.62)	4 (27%)	1–3	−0.14 (p = 0.58)	−0.70 (p = 0.004)	−0.52 (p = 0.05)
Field Guide 2 (n = 15)	1.93 (2.15)	4 (27%)	1–9	−0.26 (p = 0.31)	−**0.54 (p = 0.04)**	0.00 (p = 0.99)

SD: standard deviation; EBPAS: Evidence-based Practice Attitude Scale.

Bolded figures indicate significant correlations. N’s differ across learner assessments as not all providers completed all learner assessments.

### Participant perceptions of feasibility, acceptability and appropriateness of the JumpStart course

Fourteen of the 16 participants who completed the course filled in the post-training survey (6 specialists and 8 non-specialists). Their scores are presented in Table 3. Mean scores indicate high ratings of acceptability, appropriateness and feasibility. Higher education level was associated with higher ratings of acceptability of the course, and more positive attitudes to implementing a new evidence-based practice were associated with higher ratings of feasibility.

**Table 3.** Ratings of appropriateness, acceptability and feasibility and associations with participant characteristics (n = 14) using Spearman’s rank order correlation coefficients.

Construct	Mean (SD)	Self-rated English proficiency: rho (p-value)	Education level: rho (p-value)	Attitudes (total EBPAS score): rho (p-value)
Acceptability	4.42 (1.01)	−0.33 (p = 0.26)	0**.55 (p** **=** 0**.04)**	0.48 (p = 0.08)
Appropriateness	4.18 (0.89)	−0.38 (p = 0.18)	0.51 (p = 0.06)	0.48 (p = 0.09)
Feasibility	4.08 (0.92)	−0.43 (p = 0.12)	0.36 (p = 0.36)	0**.61 (p** **=** 0**.02)**

Maximum possible score: 5; SD: standard deviation; EBPAS: Evidence-based Practice Attitude Scale.

Responses to the open-ended question were organised thematically according to the constructs of acceptability, appropriateness and feasibility.

#### Course acceptability

Eleven of the 14 participants made explicitly positive comments on aspects related to acceptability of the course. These included reference to the helpfulness of the content in equipping providers to help families, the focus of the model on helping families improve active engagement in their daily routines, the inclusion of extensive video clips to illustrate the content and the practice opportunities given to learn the measures associated with the model, specifically, the Measure of Active Engagement and Transactional Supports (MAETS) and the Weekly Progress Form. These are exemplified in the following quotes:

I have enjoyed going through this course as it is very fitting to the needs of our clients. It has taught me a lot about Early intervention and making use of everyday activities to help the child. It has also surprised me in teaching me that it is possible to help the child learn with using household equipment which will be very helpful to our service users. (Social worker)

I thoroughly enjoyed the course. The course material was incredibly valuable and the videos helped tie everything together. I found it particularly helpful when opportunities were given for items such as the MAETS and weekly progress to be scored with accompanying guidelines. (Speech therapist)

Overall it is a good course, I will definitely be able to assist parents following this programme. (Non-specialist)

In contrast, five participants suggested challenges to course acceptability in two areas, specifically, the complexity of the language in the course for second language English speakers (2) and the length of the videos (3), as illustrated in the following quote:

Although it was not easy for me at all, because of the language, but I won’t lie, I learned a lot. (Non-specialist)

The videos, although designed to give as much information as possible, could become too long and tedious. (Non-specialist)

#### Course appropriateness

Four participants commented on positive aspects of appropriateness of the course and all felt that the course did not require any adaptations for the South African context. For example,

The course content does not require any adaptations. It is thorough and detailed. The Early Interventionist would need the required skills to deal with cultural differences and an ability to work in diverse settings, with diverse people–cultural competencies. (Social worker)

In contrast, two participants felt South African parents might have difficulty relating to the course, and one participant noted the following:

The cultural differences is another challenge that I think will also come into play, in some cultures men don’t play with children, . . . our rural areas differ greatly with rurals in America or other places abroad. I believe it is a good course but will need a lot of adaptation to suit our South African setting. (Non-specialist)

One participant suggested a South African voice-over for the slide narration to make it more ‘lively’.

#### Course feasibility

Three participants gave positive comments on feasibility of the course, describing it as ‘implementable’, having experienced ‘no technical difficulties’ while completing the course, and the course being ‘cost-effective’. With respect to feasibility challenges, one participant noted that Internet connectivity and data costs in South Africa may be a barrier to this type of training if parents were to be given the opportunity to do the ‘How-to-Guide for Families’ as follows:

If the training was to be provided to my typical parents here in the *** province, then network connection, data affordability would still be an issue. An alternative would be needed, perhaps a face-to-face approach or organized group online training. (Non-specialist)

No other feasibility challenges in completing the course were mentioned by the participants in the written post-training survey. However, in one of the bimonthly webinars, one participant mentioned that she found it easier to complete the course when it was scheduled during the workday and that all the providers went through the content together as a group. This allowed for opportunities to check understanding and discuss the content among themselves.

## Discussion

This study explored the feasibility, acceptability and appropriateness of the Autism Navigator JumpStart to Coaching in Everyday Activities course for training specialist and non-specialist NPO service providers in an evidence-based parent coaching intervention (ESI) in South Africa. The study builds on a previous study exploring another Autism Navigator web-based training (Chambers et al., 2018) and was designed to address a pressing need for building capacity in evidence-based early intervention in these low-resource service settings (Chambers et al., 2018; Franz et al., 2017, 2018). The study incorporated extensive stakeholder engagement prior to and during enrolment and an innovative web-based training course, with a local adaptation of bimonthly conference calls with each NPO’s group of providers to discuss the content as bridging strategies to maximise implementation success and (Stahmer et al., 2019), and examined inner context (provider characteristics) on utilisation and perceptions of the JumpStart course. Apart from these bridging strategies, no functional or structural changes were made at the NPO organisations which took part in the study, and the onus was on the individual providers and managers to allocate time for the training within their usual daily work routines. The findings, therefore, reflect the ability of the providers to integrate this training within their usual scope of practice in their real-world contexts, a key ingredient for sustainability. It must be noted that this study commenced about 6 months before COVID-19 lockdown restrictions were imposed in South Africa, and all the attendant stressors of that time.

We observed a 62% completion rate of the JumpStart course, which we consider reasonably successful compared to other online courses (Wang et al., 2023). This is much higher than completion rates reported for massive open online courses (MOOCs) which have been shown to vary from 0.7% to 52.1%, but are usually found to be between 8% and 15% (Jordan, 2015; Muljana & Luo, 2019; Wang et al., 2023). It is also higher than other professional CPD online courses, for example, in the field of nutrition (Stark et al., 2021) and nursing (Hosey et al., 2016), and the so-called small private online courses (SPOCs) often used for specialist continuing professional training, for example, in oncology (Vaysse et al., 2018). Our initial stakeholder engagements prior to participation and the bimonthly webinars during the training to discuss the content may have contributed to the higher completion rate. However, the completion rate is lower than that observed in our previous research with the Autism Navigator for Primary Care course of 94% (Chambers et al., 2018). This could be related to the fact that the JumpStart course was much longer than the Primary Care course of the previous study and due to some staff turnover in the NPOs during the course of this study, consisting of seven non-specialists and three specialists before they had completed the course. Although there were no differences in the proportion of specialists versus non-specialists completing the course, the fact that specialist providers were able to obtain continuing education credits with their professional bodies in South Africa could have disproportionately impacted their motivation to complete the course compared to the non-specialists. We were encouraged that none of the other provider characteristics measured in this study appeared to be related to attrition, suggesting that the course could have broad applicability to a range of service providers in South Africa.

On average, it took longer than the encouraged ideal of 3 months to complete the course and clearly at least 6 months is a more realistic expectation for most providers in this study who were working full-time. The average time to complete the course was commensurate with US providers (Wetherby, personal communication, 4 November, 2022). It was also only during the first hard lockdown in South Africa that significant numbers of enrolled providers were able to complete the course, consistent with other online courses completed during COVID (Shaikh & Asif, 2022; Wang et al., 2023). It is likely that the lockdown periods helped providers complete the course as they were unable to continue with their usual activities, and hence, it will be important to consider whether this course can be completed timeously in normal periods of daily activity. Outside of lockdown, it seemed that providers had difficulty implementing self-guided training into their busy workdays, despite overt managerial buy-in and support for the study and the training. One non-specialist provider reported that completing the course during the workday as a group with other providers in the organisation was the most satisfactory way to do the training, rather than individually as a self-paced course. This arrangement allowed for discussion of new or difficult content to enhance understanding. It would also have allowed for time during the workday to complete the training and access to free Wi-Fi or data through the organisations rather than relying on personal Wi-Fi or data at home. This adaptation could be key for NPOs to help groups of providers complete the course timeously. The fact that providers were so successful in completing the course during lockdown highlights the versatility and reach of the course during a global healthcare crisis when typical in-person clinical training could not take place.

It was encouraging that no provider characteristics measured in this study were related to time taken to complete the course. Specifically, English proficiency, education level, computer access, prior experience with online training and attitudes to implementing an evidence-based practice were not related to the time taken to complete the course among those who did manage to complete it. However, provider characteristics were related to the number of attempts needed to achieve a passing score of 80% or higher on some learner assessments. Specifically, those with lower self-ratings of English proficiency needed more attempts to pass Guide Books 3 and 4 learner assessments, while those with lower levels of education (i.e. non-specialists) needed more attempts to pass the caregiver coaching (Field Guide 2) learner assessment. As described in the Supplemental Material, Guide Book 3 covered content related to active engagement and transactional supports, and Guide Book 4 covered material on challenging behaviour (understanding the function of challenging behaviour and implementing a positive behavioural support plan). It is possible that these two sections contained technical jargon making these sections less accessible for those less proficient in English. Field Guide 2 covered content related to caregiver coaching theory and practice, which may have been more accessible to those with clinical training and prior clinical experience. These results suggest that the JumpStart course may not be universally applicable to all providers in South Africa and that the course is more feasible for those with better English proficiency and clinical specialist providers. However, providers from a diverse range of backgrounds were able to complete the course with sufficient support.

Similar to our previous study (Chambers et al., 2018), the majority of provider participants rated the Autism Navigator JumpStart course as highly acceptable, appropriate and feasible in their NPO settings and commented that it addressed important knowledge gaps for them. Comments specifically highlighted the helpfulness of the content in equipping providers to help the families they usually serve, the focus of the model on helping families improve active engagement in their daily routines, a key ingredient of ESI and the inclusion of the extensive library of video clips to illustrate the content (although these were also sometimes described as too long). The web-based, self-paced nature of the course was especially liked by most providers, and leant itself to continued engagement and completions during COVID-19 lockdown periods. Ratings of acceptability were higher among those with higher education levels, and ratings of feasibility were higher among those with more positive attitudes to implementing a new evidence-based practice. Thus, higher education levels and more positive attitudes to implementing a new practice may have mediated the benefit of the course for providers.

Although not related to the training itself, an important observation were the contrasting views on cultural appropriateness of the ESI intervention itself. Two providers felt that some activities they viewed in the videos were not appropriate to South African families, such as a father playing with their child. However, most providers felt the intervention could be implemented in any cultural context and that by working with children in their typical daily activities, as opposed to more artificial therapeutic play environments, providers are naturally able to make the intervention culturally appropriate for families. This is indeed the intention of the ESI model, and by using the families’ natural daily routines and activities, it is possible to tailor the intervention to any cultural context. However, it is true that being able to see how this implementation looks with South African families would make the training more relatable, for both South African providers and families. The inclusion of additional video clips with South African families would likely make the training more acceptable and appropriate for the South African context.

There are a number of limitations in this study. One is the large number of measures used with a small pool of participants. Results from nonparametric correlations were only likely to detect large effects and thus some effects may have been masked in the study. In addition, perceptions of feasibility, acceptability and appropriateness were only obtained from those who completed the course, which could have resulted in a positive bias. The post-training survey yielded valuable information in the study, but there was a richer depth to the discussions in the webinars during the training. These data may have yielded greater insights into the experience of providers with the JumpStart course had it been included in the analyses. In addition, it must be noted that the programme developers assisted in the study by covering the cost of the participant’s seats in the JumpStart course and supporting the first author financially in her role to support the providers. While all care was taken to minimise any potential influences of this arrangement, the findings should be interpreted with this in mind. Finally, this study did not assess the ability of the trained providers to implement the ESI intervention they learned about in the course with families. This is an essential next step for future research as the value of the training cannot be truly evaluated without knowing whether it leads to accurate fidelity of implementation with measurable effects on caregivers and their children.

There are no established benchmarks to determine feasibility of online professional development courses in the field of autism. We concluded that our completion rate of 62% demonstrates success and viability. If we had required course completion in 3 months, our completion rate would have been much lower. A much lower completion rate, such as 20% in less time, would still be feasible and may be considered viable and acceptable, if it is at or above industry standards (e.g. 8%–15% for MOOCs). Contributions of this study, in the context of other feasibility studies, are to characterise how bridging strategies, allowance for more time and demographic variables can improve completion rates. Recent research has offered frameworks to identify factors related to completion, retention and dropout, such as social engagement and connection, course design and difficulty, instructor feedback, interaction in the online learning community and institutional support (Karunarathne et al., 2023; Muljana & Luo, 2019; Shaikh & Asif, 2022; Wang et al., 2023). The variability in feasibility studies offers valuable learning opportunities for the field. Future research is needed to better understand what predicts successful indicators, such as completion and engagement, and to document improvement strategies.

## Conclusion

Training in evidence-based intervention for young children with autism is imperative for providers in NPOs dedicated to assisting families. The web-based Autism Navigator JumpStart to Coaching in Everyday Activities and accompanying group webinars was considered a potentially valuable training tool through stakeholder engagement prior to this study. Out of 26 providers enrolled in the study, 62% were able to complete the course over an average of 5.58 months. No provider characteristics measured in this study were associated with attrition, nor time to complete the course. However, better English proficiency, higher education levels and more positive attitudes to implementing an evidence-based intervention were associated with the ability to pass some learner assessments with fewer attempts, and to subjective ratings of course feasibility and acceptability. Adaptations in the training delivery, such as completing it as a group within organisations, and the inclusion of South African video clips in the video libraries may be potential facilitators for training completion and appropriateness within the South African context.

## Supplemental Material

sj-docx-1-aut-10.1177_13623613231223784 – Supplemental material for Feasibility of the Autism Navigator® JumpStart to Coaching in Everyday Activities course in South Africa Supplemental material, sj-docx-1-aut-10.1177_13623613231223784 for Feasibility of the Autism Navigator® JumpStart to Coaching in Everyday Activities course in South Africa by Nola Chambers, Petrus J de Vries and Amy M Wetherby in Autism
