# Supplementary material for: Feasibility of the Autism Navigator® JumpStart to Coaching in Everyday Activities course in South Africa
Source: Autism. 2024 Feb 8;28(9):2232–43. doi: 10.1177/13623613231223784 (PMC11395170; doi:10.1177/13623613231223784)
Supplement: sj-docx-1-aut-10.1177_13623613231223784 – Supplemental material for Feasibility of the Autism Navigator® JumpStart to Coaching in Everyday Activities course in South Africa [file sj-docx-1-aut-10.1177_13623613231223784.docx]

**Supplemental Material:**

**Description of the ESI Model and Autism Navigator JumpStart to Coaching in Everyday Activities Course**

**Theoretical background**

The Early Social Interaction (ESI) Model is a comprehensive early intervention approach developed for toddlers with autism spectrum disorder (ASD) and their families incorporating evidence-based active ingredients consistent with NDBIs (Schreibman et al., 2015; Tiede & Walton, 2019). This supplemental information provides details on the ESI model, SCERTS curricular-based assessment and intervention model, evidence base for ESI, and the overall components of the JumpStart to Coaching in Everyday Activities online course to train professionals on implementation of the ESI model.

**ESI Model**

The ESI model incorporates the following features:

**1) Developmental framework to prioritize child outcomes.** ESI uses SCERTS, a manualized curricular-based assessment and intervention framework, to identify goals and objectives and monitor progress (Prizant, Wetherby, Rubin, Laurent, & Rydell, 2006). The acronym “SCERTS” refers to Social Communication (SC), Emotional Regulation (ER) and Transactional Support (TS), which are the primary developmental dimensions targeted to support the development of children with ASD and their families. The SCERTS curricular-based assessment includes parent report and observation forms administered in the child’s home with the family to identify high priority goals and objectives. Assessments are updated monthly and quarterly. The SC and ER domains delineate specific, measurable goals and objectives for the child and are organized by communication stage, beginning with children who are in the Social Partner Stage, before the development of any words. The TS domains delineate specific, measurable teaching strategies for the parent or other communicative partners and include teaching strategies and learning supports that are selected to help the child meet his/her individualized goals and objectives.

The ESI model has developed a “layer cake” of evidence-based strategies and supports that parents learn to implement activity by activity in a variety of activities across 8 categories of everyday activities: play with people (without objects), play with props (large objects), meals and snacks, caregiving, play with toys (small objects), family chores, sharing books, and transitions between activities. ESI uses a “layer cake” of 13 evidence-based strategies and supports, adapted from the SCERTS developmental framework and incorporating behavioural techniques that reflect the common features of NDBIs delineated by Schreibman et al. (2015). Like making a “layer cake”, it’s important to set up the first layer before adding the second and third layers. Because they are cumulative, later supports rely on earlier supports being in place.

Layer 1 builds a shared agenda for the parent and child with 5 strategies:

- A motivating activity is something a child wants to do that includes interesting and preferred materials. Activities such as hand washing are necessary but may not be motivating. Knowing that a fun activity is coming after a necessary activity is one way to make a necessary activity motivating. Adding interesting materials or other supports for a shared agenda can also help make necessary activities more interesting or sensible for your child and might even make them more fun.
- A productive role lets a child know exactly what to do and that they are expected to use materials in an appropriate way and actively attend and participate. Roles can be as simple as turning a page, dropping clothes into a hamper from the dryer, or gathering materials for a painting activity.
- Predictability of the activity lets a child know what is coming next by making the beginning, middle, and end of each activity clear to your child, and what’s happening next.
- Positioning so that a parent is close by, at a child’s eye level, and face-to-face to make it easier for your child to notice you, look at you, and interact with you.
- Follow a child’s attentional focus means noticing and talking about what a child is paying attention to, doing, or experiencing. Asking a child to shift their attention to something else is placing more burden on the child’s social attention and can interfere with establishing a shared agenda and learning.

Layer 2 builds Supports for Social Reciprocity using 4 strategies:

- Promote child initiation by setting up a reason for a child to communicate, then waiting. When you ask a child to say a word they practice responding- not initiating communication. Natural pauses encourage a child to practice communicating their own ideas.
- Balance turns so that you take no more than 1½ turns for each of a child’s turns. Keep in mind that your turn can be a comment, gesture or action that supports a child to take a turn next. A child may also take turns using actions, gestures, or words.
- Natural reinforcers such as offering help, comfort, a favorite snack or toy, shared enjoyment, or shared interest naturally reward a child’s communication. Saying “good talking!” has no connection with the intention a child is trying to communicate and therefore does not help the child learn that their words have power and meaning.
- Clear messages to ensure comprehension make it clear to a child that you expect them to listen to and act on your words. Gestures or other contextual support are offered to ensure that a child understands your message. Language that is optional for a child to listen to may give the impression that the language of others can be “tuned out” or disregarded.

Layer 3 builds Supports for Better Skills to promote social communication development using the following 4 strategies:

- Model language, play, and interaction to help a child know what they could say or do. Model language just above a child’s level, and use their perspective when possible, “as they would say it if they could”. Providing a model of language or behaviour builds better skills by example and by giving the turn back to the child. Giving directions or asking questions can provide a more limited response and may not build better skills.
- Extend the activity, roles, and transitions to build on play, interaction, and connectedness. “Stretching” engaged time spent in activities and expanding roles your child can take on during transitions can add up quickly to more engaged time per week.
- Adjust expectations and demands by offering more support when a child is struggling, and increasing expectations when things are going well to keep the child moving forward. Over time, you should be able to do less of the “work” and shift more responsibility to the child to keep interaction and communication going.
- Balance interaction and independence by supporting interaction but also building independence with an eye toward what a child needs to learn in a group setting to prepare for the skills needed in preschool and kindergarten classrooms.

**2) Systematic instruction using evidence-based strategies.** Children with ASD can learn in everyday activities when learning opportunities are structured and systematic techniques are used to foster active engagement. ESI incorporates systematic instruction using evidence-based behavioural and developmental strategies for toddlers. ESI uses well established behavioural strategies that are evidence-based NDBIs (Schreibman et al., 2015) including using antecedent-behaviour-consequence contingency in learning trials, environmental arrangement and support to promote child-initiated teaching trials or episodes, prompting and prompt fading, and natural reinforcers. Naturalistic activities and developmental strategies are used to promote self-initiated spontaneous behaviour that will generalize to new activities and settings. ESI uses environmental arrangement, predictability, scaffolding with waiting and modelling, and then, if needed verbal and or nonverbal prompting with immediate fading, so the child does not become prompt dependent.

Parents learn to use intervention strategies matched to the priority objectives within daily activities to increase opportunities for teaching and learning whenever the activity occurs. Ongoing monitoring with corresponding adjustments in programming is based on observational data collected on parents’ use of strategies and child targets within activities during each session and in monthly and quarterly updates using naturalistic home observations without the coach.

**3) Collaborative coaching to support parent learning and generalization.** Individual intervention sessions are organized to build parent capacity to engage in their children’s learning and include updating session plan, practicing supports and strategies in 3-5 different activities, problem solving, and planning for ongoing intervention between sessions. A four-step collaborative coaching model based on adult learning research is used: 1) identify what works, with direct teaching if needed; 2) guided practice with parent in an active role but guided by the coach with specific feedback; 3) parent-led practice and reflection with specific feedback; and 4) interventionist backs out for parent independence in running the activity, problem-solving, reflecting, and making adjustments. The interventionist coaches the parent in each new activity at the first level and moves to level 4 as quickly as possible to promote parent competence, confidence, and independence. Coaching in a variety of everyday activities promotes generalization of parent learning so the parent can support the child’s learning throughout the day as planned or as opportunities arise.

**4) Family-centred approach.** A family-centred approach addresses the family’s needs, concerns, and priorities during the assessment and intervention process and is required in the IDEA Part C governing early intervention. Respecting family members’ perceptions, priorities, and preferences, developing active participatory and relational partnerships and moving from building consensus to building capacity are key components of an effective family-centred program. Families are more involved in the achievement of goals when they have been stakeholders in their development.

**5) Learning in natural environments.** Natural environments are the context for early intervention in IDEA Part C and are defined as the everyday activities, routines, and settings typical for any family including their home, childcare, and community locations such as the park or grocery store. Everyday activities such as mealtime, play, caregiving, and family chores provide authentic opportunities to embed teaching of intervention targets that are functional to the activity and therefore naturally support acquisition and generalization of the skills. Individual coaching of families to embed evidence-based intervention strategies throughout the day requires interventionist and parent consideration of the activity sequence, ease of strategy use, and frequency of learning opportunities within various activities. Group parent education meetings and playgroups including families of young typical children also offer a natural environment for family members of children with ASD to get information and support.

**6) Intensity needed for children with ASD.** The intensity needed for children with ASD is achieved through the integration of the core features of ESI. Parents partner with professionals to plan an individualized, developmentally-sensible intervention program using SCERTS to address the impact of the child’s autism symptoms on learning. Professionals coach parents on how to competently and systematically use intervention strategies throughout the day in typical activities where the skills are meaningful for the child. This process of embedding strategies within everyday activities is designed to support parent implementation of 25 hours/week of active child engagement. While the intensity of intervention necessary to provide optimal outcomes is not yet determined for infants and toddlers at risk for ASD, research suggests that more time spent in active, positive engagement results in better outcomes for preschoolers. A minimum of 25 hours per week of active engagement in intervention has been recommended as soon as children are suspected of having ASD. The ESI parent-implemented intervention provides a way to maximize intensity of intervention and reduce professional time.

**Evidence Base for ESI**

Evidence for ESI in toddlers was demonstrated in a multisite randomized controlled trial by Wetherby and colleagues (2014) of 82 toddlers with ASD and their families comparing two parent-implemented intervention conditions for 9 months each: (a) individual-ESI offered in two to three weekly sessions at home to teach parents how to embed strategies to support social communication skills for 25 hr a week within everyday routines, activities, and places and (b) group-ESI with an information, education, and a support group offered once a week (Wetherby et al., 2014). After 9 months of intervention, children in both conditions showed significant improvement on all three composites of the Communication and Symbolic Behavior Scales Behavior Sample (Wetherby & Prizant, 2002), but children in individual ESI made significantly greater gains on the social composite (Time × Condition: F(1, 71.79) = 4.14, p = .04, Hedge’s g = 0.48). On the Mullen Scales of Early Learning, children in both group and individual conditions showed significant improvements on receptive and expressive language scales, but children in individual ESI showed significantly greater gains in receptive language, F(1, 74.45) = 7.46, p = .008, Hedge’s g = 0.58. On the Vineland Adaptive Behavior Scales, children in both conditions showed significant improvements in communication, but children in individual-ESI showed significantly greater gains, F(1, 75.72) = 8.76, p = .004, Hedge’s g = 0.69. On the Autism Diagnostic Observation Schedule, children in both conditions showed a significant decrease in symptom severity on Social Affect.

In addition to these improvements in distal outcomes, Wetherby and colleagues also reported changes in a proximal measure of active engagement in the RCT sample (Wetherby et al., 2018). Again, both groups improved significantly on the Measure of Active Engagement (MAE) during 0-3 months and 3-6 months of the intervention. However, the individual ESI treatment group improved significantly more in the 0-3 month treatment window. In addition, these greater gains in MAE scores were related to higher scores in those distal outcome measures at the end of the 9-month treatment condition as reported in the original RCT (Wetherby et al., 2014).

Finally, Guthrie and colleagues recently reported on the findings of the crossover design of the original 82 RCT participants to specifically examine the impact of timing of the intervention (Guthrie et al., 2023). A significant treatment timing effect was defined as a significantly greater change during Condition 1 (18-27 months) than Condition 2 (27-36 months) for Individual-ESI and no greater change during Condition 1 than Condition 2 for Group-ESI, in order to rule out maturation effects. Significantly greater change during Condition 1 than Condition 2 for both Individual- ESI and Group-ESI was not considered a treatment timing effect, as such change could be due to maturation. Results revealed that children who received Individual-ESI in Condition 1 at 18 months showed significantly greater gains during the 9-month treatment period, compared to children who received Individual-ESI during Condition 2 at 27 months across several outcome domains including the social composite of the CSBS, receptive language on the Mullen, and three subscales of the Vineland (Receptive, Expressive and Daily Living). Planned comparisons were non-significant for Group-ESI for these outcome variables, suggesting that maturation effects cannot explain these differences and significant timing effects were specific to Individual-ESI.

In summary, individually delivered ESI has been shown to results in superior proximal and distal outcomes over and above group-based ESI. In addition, earlier initiation of individual ESI (18 months) resulted in greater beneficial changes than individual ESI initiated at 27 months.

**Components and Content of the Autism Navigator JumpStart to Coaching in Everyday Activities Course**

The Autism Navigator JumpStart to Coaching in Everyday Activities is a professional development online course designed to distil the essential ingredients of ESI into a self-guided web-based course for interprofessional training embedded within a process of continuing professional development with an implementation science focus. In terms of content, the JumpStart course consists of two major sections. The first is the Autism Navigator How-To Guide for Families, which was designed for families as a resource to learn about active engagement goals, caregiver transactional support, and how to implement these in a variety of everyday activities. In addition to the How-To-Guide, there are additional modules specifically for providers implementing the caregiver coaching with families. These two modules or Field Guides focus on building consensus with families on the early signs of autism and how to coach families on how to embed evidence-based strategies into their everyday activities to support their child’s learning and development. The components of the JumpStart course are listed in Table 1.

**Table 1: Content sections of the Autism Navigator JumpStart course**

| **Component** | **Brief description of content** |
| --- | --- |
| *Autism Navigator How-To-Guide for Families* | |
| Guide Book 1: Social Communication Milestones and how Autism Impacts Development | Includes instruction on key social communication milestones in infants and toddlers in 5 developmental domains: Emotional Regulation, Play, Social Connectedness, Language and Self-Directed Learning;  • How the early signs of autism unfold and impact development and learning in these 5 developmental domains  • Importance of early gestures and what are the 16 gestures that all children should have by 16 months  • How to differentiate late bloomers from late talkers with persisting language problems  • Importance of preventing the secondary impact of autism on cognitive development and behavioral challenges and how to promote active engagement in young children with autism |
| Guide Book 2: Collaborating to Make Early Intervention Work for You | Includes instruction on the critical role of the family in detecting the early signs of autism and family perspectives on learning their child may have autism and the impact on the family when early signs are missed  • Concerns about labelling a child and having difficult conversations about autism  • Professional perspectives on how to build consensus on the early signs and promote ongoing collaborations  • What are early intervention services and how a family can get started in early intervention |
| Guide Book 3: Getting Started with Early Intervention Right Away | Includes instruction on why learning in everyday activities in the natural environment is so important to achieve the intensity needed for young children with autism  • How to create learning opportunities in everyday activities at home and in the community  • The components of active engagement to help decide which targets are priority intervention outcomes for your child  • Evidence-based intervention supports you can use in everyday activities to promote active engagement that are organized into 3 layers like a layer cake |
| Guide Book 4: Addressing Challenging Behaviors | Includes instruction on why children with autism have challenging behaviors  • How to understand the function of challenging behaviors  • How to develop and implement a positive behavior support plan in partnership with families |
| Library of Change with Intervention | Library of edited video clips showing change with intervention from 18-36 months of age for 8 toddlers with autism and their families |
| Library of Everyday Activities | Library of 200 video clips of different families coached in ESI interacting with their toddlers with autism in a variety of activities in 8 categories of everyday activities |
| *Additional Modules for Providers* | |
| Field Guide 1: Having Difficult Conversations about the Early Signs of Autism | Includes instruction on how to identify the early signs of autism; how to communicate to build relationships with families; how to implement family-centered services and supports; and how to collaborate to build consensus with families |
| Field Guide 2: Coaching in Everyday Activities | Includes instruction on how to collaborate and build consensus with families regarding intervention outcomes and strategies; how to coach families to embed evidence-based intervention strategies and supports in their everyday activities; and how to use Autism Navigator tools to monitor meaningful progress to determine how well the intervention is working and whether program adjustments are needed |

**Course components and design**

The Jumpstart course (and other courses within the Autism Navigator suite of courses) are designed using principles of adult learning (Trivette, Dunst, Hamby, & Herin, 2009) and includes manualization with explicit procedures illustrating the intervention, supported by coaching and feedback of the personnel, to improve fidelity (Dunst, 2017). It expands beyond self-directed resources and modules to encompass a complete system of professional development with a multiple component, learner-oriented approach. It includes varying delivery formats, such as interactive videos, webinars, just-in-time resource guides and rubrics. The web-based system serves as a reference for providers at any time to review, explore resources, and problem-solve options for intervention through review of the video libraries. Finally, it includes a system for building communities of practice for sharing experiences and discussing implementation strategies, all essential features of successful professional development (Myers, Brown, & Parigian, 2022; Pianta, Mashburn, Downer, Hamre, & Justice, 2008). Autism Navigator convenes monthly webinars for participants enrolled in the JumpStart course to discuss what they are learning. These gatherings are designed to increase learner engagement with course content and related tools and include interactive discussions based on illustrative video exemplars.

**References**

Dunst, C. J. (2017). Procedures for Developing Evidence-Informed Performance Checklists for Improving Early Childhood Intervention Practices. *Journal of Education and Learning*, *6*(3), 1–13. https://doi.org/10.5539/jel.v6n3p1

Guthrie, W., Wetherby, A. M., Woods, J., Schatschneider, C., Holland, R. D., Morgan, L., & Lord, C. E. (2023). The earlier the better : An RCT of treatment timing effects for toddlers on the autism spectrum. *Autism*, 13623613231159152. https://doi.org/10.1177/13623613231159153

Myers, C. T., Brown, S., & Parigian, A. (2022). Exploration of a Short-term Learning Community Focused on Evidence-based Occupational Therapy Interventions for Children and Youth with Autism. *Journal of Occupational Therapy Education*, *6*(3). https://doi.org/10.26681/jote.2022.060313

Pianta, R. C., Mashburn, A. J., Downer, J. T., Hamre, B. K., & Justice, L. (2008). Effects of web-mediated professional development resources on teacher-child interactions in pre-kindergarten classrooms. *Early Childhood Research Quarterly*, *23*(4), 431–451. https://doi.org/10.1016/j.ecresq.2008.02.001

Prizant, B., Wetherby, A., Rubin, E., Laurent, A., & Rydell, P. (2006). *The SCERTS model: A comprehensive educational approach for children with autism spectrum disorders, Vol. 1.* Paul H Brookes Publishing Co.

Schreibman, L., Dawson, G., Stahmer, A. C., Landa, R., Rogers, S. J., McGee, G. G., … Halladay, A. (2015). Naturalistic Developmental Behavioral Interventions: Empirically Validated Treatments for Autism Spectrum Disorder. *Journal of Autism and Developmental Disorders*, *45*, 2411–2428. https://doi.org/10.1007/s10803-015-2407-8

Tiede, G., & Walton, K. M. (2019). Meta-analysis of naturalistic developmental behavioral interventions for young children with autism spectrum disorder. *Autism*, *23*(8), 2080–2095. https://doi.org/10.1177/1362361319836371

Trivette, C. M., Dunst, C. J., Hamby, D. W., & Herin, C. E. O. (2009). Characteristics and Consequences of Adult Learning Methods and Strategies, *2*(1).

Wetherby, A. M., Guthrie, W., Woods, J., Schatschneider, C., Holland, R. D., Morgan, L., & Lord, C. (2014). Parent-Implemented Social Intervention for Toddlers With Autism: An RCT. *Pediatrics*, peds.2014-0757. https://doi.org/10.1542/peds.2014-0757

Wetherby, A. M., Woods, J., Guthrie, W., Delehanty, A., Brown, J. A., Morgan, L., … Lord, C. (2018). Changing Developmental Trajectories of Toddlers With Autism Spectrum Disorder: Strategies for Bridging Research to Community Practice. *Journal of Speech Language and Hearing Research*, *61*(11), 2615. https://doi.org/10.1044/2018_JSLHR-L-RSAUT-18-0028

Wetherby, A., & Prizant, B. (2002). *Communication and Symbolic Behavior Scales: Developmental Profile*.Brookes Publishing.
